# Effects of different fluid management on lung and kidney during pressure‐controlled and pressure‐support ventilation in experimental acute lung injury

**DOI:** 10.14814/phy2.15429

**Published:** 2022-09-06

**Authors:** Eduardo Butturini de Carvalho, Ana Carolina Fernandes Fonseca, Raquel Ferreira Magalhães, Eliete Ferreira Pinto, Cynthia dos Santos Samary, Mariana Alves Antunes, Camila Machado Baldavira, Lizandre Keren Ramos da Silveira, Walcy Rosolia Teodoro, Marcelo Gama de Abreu, Vera Luiza Capelozzi, Nathane Santanna Felix, Paolo Pelosi, Patrícia Rieken Macêdo Rocco, Pedro Leme Silva

**Affiliations:** ^1^ Laboratory of Pulmonary Investigation, Institute of Biophysics Carlos Chagas Filho Federal University of Rio de Janeiro Rio de Janeiro RJ Brazil; ^2^ University of Vassouras Vassouras RJ Brazil; ^3^ Department of Pathology, School of Medicine University of São Paulo São Paulo Brazil; ^4^ Pulmonary Engineering Group, Department of Anaesthesiology and Intensive Care Therapy, Technische Universität Dresden University Hospital Carl Gustav Carus Dresden Germany; ^5^ Department of Intensive Care and Resuscitation, Anesthesiology Institute Cleveland Clinic Cleveland Ohio USA; ^6^ Department of Outcomes Research, Anesthesiology Institute Cleveland Clinic Cleveland Ohio USA; ^7^ Department of Surgical Sciences and Integrated Diagnostics University of Genoa Genoa Italy; ^8^ Anesthesia and Critical Care, San Martino Policlinico Hospital IRCCS for Oncology and Neurosciences Genoa Italy

**Keywords:** acute lung injury, fluid therapy, hemodynamics, immunofluorescence, immunohistochemistry, molecular biology, pressure‐support ventilation

## Abstract

Optimal fluid management is critical during mechanical ventilation to mitigate lung damage. Under normovolemia and protective ventilation, pulmonary tensile stress during pressure‐support ventilation (PSV) results in comparable lung protection to compressive stress during pressure‐controlled ventilation (PCV) in experimental acute lung injury (ALI). It is not yet known whether tensile stress can lead to comparable protection to compressive stress in ALI under a liberal fluid strategy (LF). A conservative fluid strategy (CF) was compared with LF during PSV and PCV on lungs and kidneys in an established model of ALI. Twenty‐eight male Wistar rats received endotoxin intratracheally. After 24 h, they were treated with CF (minimum volume of Ringer's lactate to maintain normovolemia and mean arterial pressure ≥70 mmHg) or LF (~4 times higher than CF) combined with PSV or PCV (V_T_ = 6 ml/kg, PEEP = 3 cmH_2_O) for 1 h. Nonventilated animals (*n* = 4) were used for molecular biology analyses. CF‐PSV compared with LF‐PSV: (1) decreased the diffuse alveolar damage score (10 [7.8–12] vs. 25 [23–31.5], *p* = 0.006), mainly due to edema in axial and alveolar parenchyma; (2) increased birefringence for occludin and claudin‐4 in lung tissue and expression of zonula‐occludens‐1 and metalloproteinase‐9 in lung. LF compared with CF reduced neutrophil gelatinase‐associated lipocalin and interleukin‐6 expression in the kidneys in PSV and PCV. In conclusion, CF compared with LF combined with PSV yielded less lung epithelial cell damage in the current model of ALI. However, LF compared with CF resulted in less kidney injury markers, regardless of the ventilatory strategy.

## Graphical abstract

It is not yet known whether tensile stress can lead to comparable lung protection than compressive stress in acute lung injury (ALI) under a liberal fluid (LF) strategy. Thus the interaction between fluid and ventilator strategies was evaluated. Normovolaemia combined with pressure‐support ventilation yielded less lung epithelial cell damage compared with LF strategy in the current model of ALI. However, liberal compared with conservative fluid strategy resulted in less kidney injury markers, regardless of the ventilatory strategy.

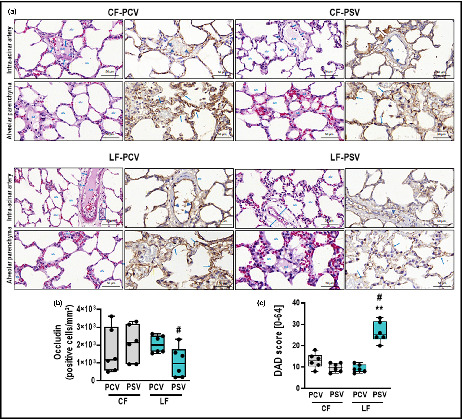

## 1 INTRODUCTION

Ventilatory support and fluid therapy are cornerstones during the management of acute lung injury (ALI) (Cruz et al., 2018; Vieillard‐Baron et al., 2016). Respiratory insufficiency requires the implementation of mechanical ventilation (MV), which in turn may worsen ventilator‐induced lung injury (VILI). Overall, 60% of critically ill patients are also hemodynamically unstable, often requiring a high intake of intravenous fluids to restore tissue perfusion (Cruz et al., 2018; Gattinoni et al., 2010; Huppert et al., 2019; Marini & Rocco, 2020; Mekontso Dessap et al., 2016). However, little is known concerning interactions between fluids and the ventilatory strategy.

Assisted spontaneous ventilation can reduce VILI compared with controlled MV in preclinical (Magalhães et al., 2018; Pinto et al., 2020; Saddy et al., 2010) and clinical (van Haren et al., 2019) studies; the latter show increased ventilation‐free days and fewer days in the intensive care unit (Saddy et al., 2010, 2013; Santos et al., 2017; van Haren et al., 2019). However, due to cardiopulmonary interaction, assisted spontaneous breathing can increase lung perfusion (due to higher right ventricle preload and lower afterload) and transvascular filtration pressure, both of which may worsen lung edema (Vieillard‐Baron et al., 2016; Yoshida et al., 2017). In addition, lung edema may increase further if tight junction connections, as occludin, which are constitutive in epithelial and endothelial structural cells, are lost during the stretch movements (Cavanaugh Jr et al., 2001). During assisted spontaneous ventilation such as pressure‐support ventilation (PSV), pleural pressure decreases, leading to tensile stress (Silva et al., 2022), whereas during pressure‐controlled ventilation (PCV), a positive increase in pleural pressure is observed, resulting in compressive stress (Silva & de Abreu, 2018). The difference between tensile and compressive stresses has been studied both in vitro (Bachofen et al., 1987; Tschumperlin et al., 2000) and in vivo (Roan & Waters, 2011). Under normovolemia and protective ventilation, tensile stress in the lung during PSV leads to comparable pulmonary protection to compressive stress during PCV in experimental ALI (Pinto et al., 2020). We hypothesized that the tensile stress during PSV would be protective under conservative fluid (CF) strategy, as compared to compressive stress during PCV, but not under a liberal fluid strategy (LF). We compared the interaction of CF and LF during PSV and PCV on lungs and kidneys in an established ALI model.

## 2 MATERIALS AND METHODS

### 2.1 Study approval

This study was approved by the Animal Care and Use Committee (CEUA: 038–18) of the Health Sciences Center, Federal University of Rio de Janeiro. All animals were treated in compliance with the *Principles of Laboratory Animal Care* by the National Society for Medical Research and the US National Academy of Sciences *Guide for the Care and Use of Laboratory Animals*. The current study followed the guidelines of the *Animal Research: Reporting of* In Vivo *Experiments* (Percie du Sert et al., 2020). Conventional animals were housed at a controlled temperature (23°C) in a controlled light–dark cycle (12–12 h), with free access to water and food.

### 2.2 Animal preparation

Beginning at 8:00 a.m., 28 male Wistar rats (body weight, 389 ± 40 g, 10–12 weeks old) were anesthetized consecutively by inhalation of isoflurane 1.5%–2.0% (Isoforine; Cristália) during spontaneous breathing and underwent intratracheal instillation of *Escherichia coli* lipopolysaccharide (LPS, 9.6 × 10^6^ endotoxin units/ml) (Merck Millipore), suspended in 0.9% saline solution (total volume 200 μl) to induce ALI (Magalhães et al., 2018; Silva et al., 2013). After full recovery from anesthesia and a 24‐h observation period, in the early morning (around 8:00 a.m.) the animals received diazepam (10 mg/kg) and ketamine (100 mg/kg, i.p.) intraperitoneally (i.p.), and tracheostomy was performed after local anesthesia (0.4 ml of 1% lidocaine) in the ventral neck midline. Anesthesia was maintained by a syringe pump (Digipump, Digicare Animal Health) delivering an constant rate infusion of ketamine and midazolam (50 and 2 mg/kg/h, respectively) intravenously (i.v.) through a tail vein catheter (Jelco 24G; Becton Dickinson). An 18G catheter (Arrow International) was inserted in the right internal carotid artery to measure continuous mean arterial pressure (MAP). The level of anesthesia was titrated according to MAP levels during experiment. Heart rate and body temperature were monitored continuously (LifeWindow 6000 V; Digicare Animal Health). Body temperature was maintained at 37.5 ± 1°C with a heating bed (EFF 421, INSIGHT). Esophageal pressure (*P*_es_) was measured using a 30‐cm water‐filled catheter with side holes at the tip (PE205, Becton Dickinson) passed into the esophagus and connected to a pressure transducer (UT‐PL‐400; SCIREQ). Correct positioning was assessed by the occlusion test (Baydur et al., 1982). Four animals were subjected to intratracheal instillation of LPS and were used as nonventilated controls for molecular biology analyses after 24 h.

### 2.3 Experimental protocol

**FIGURE 1:**
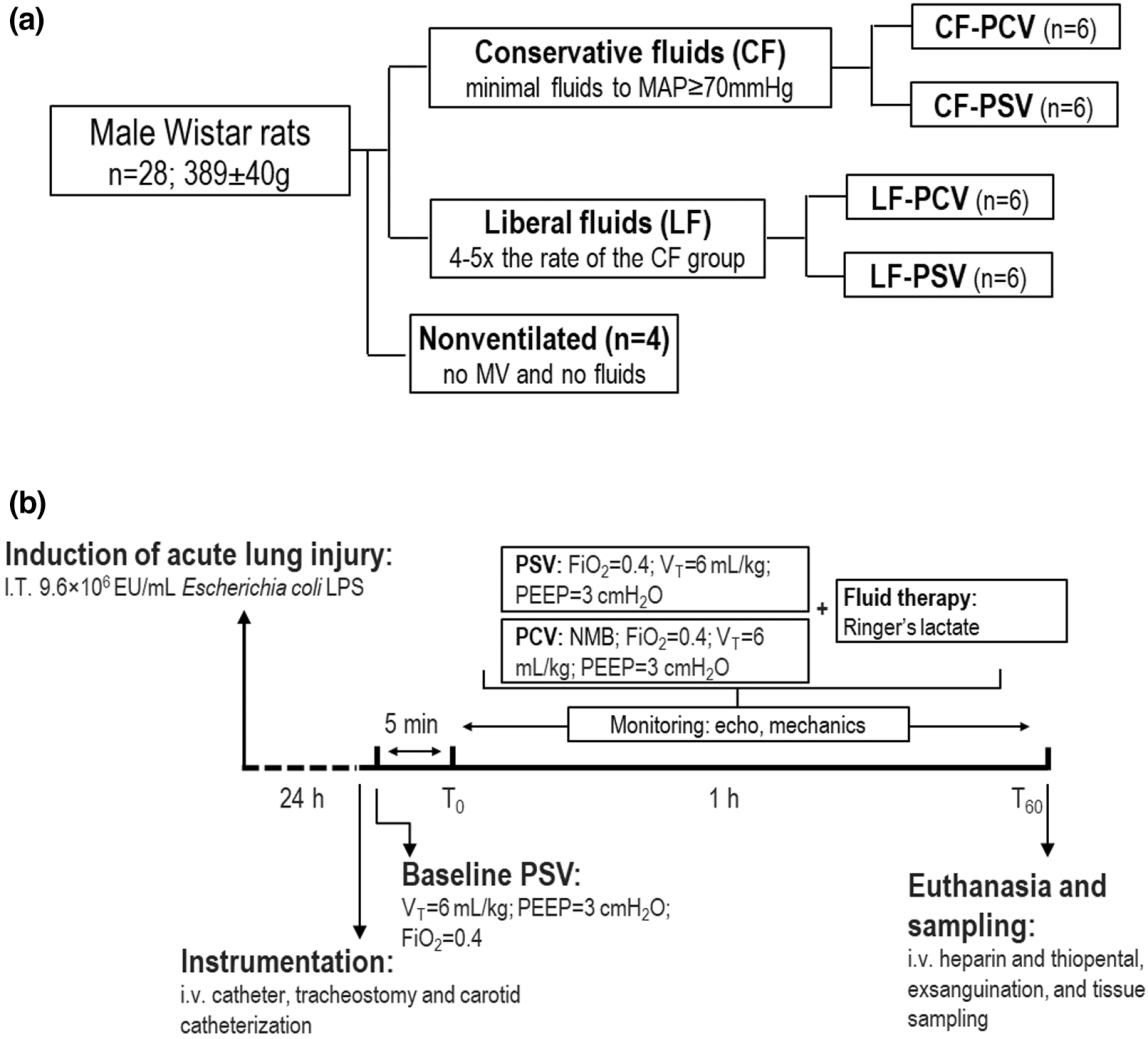
Experimental design and temporal evolution. (a) Experimental design. The CF regimen was defined as the minimal amount of Ringer's lactate to maintain mean arterial pressure (MAP) ≥70 mmHg. The LF regimen was defined as ~4–5 times higher fluids than in the CF groups. (b) Temporal evolution. CF, conservative fluid; Echo, echocardiography; FiO_2_, inspired fraction of oxygen; i.v., intravenous; LF, liberal fluid; MV, mechanical ventilation; PEEP, positive end‐expiratory pressure; V_T_, tidal volume.

After animal preparation, lungs were ventilated mechanically with Servo‐i (MAQUET) via PCV or PSV (flow triggering) during which a researcher (E.B.C.) adjusted the driving pressure to V_T_ ≈ 6 ml/kg (PEEP = 3 cmH_2_O and FiO_2_ = 0.4). As PCV inherently requires neuromuscular blockade, animals in this group were paralyzed by intravenous administration of pancuronium bromide (1 mg/kg; Cristália). Pilot studies were performed in the CF strategy group to estimate the minimal amount of Ringer's lactate (B. Braun) required to maintain normovolemia (evaluated using echocardiography) and MAP ≥70 mmHg for 1 h in experimental ALI. The mean cumulative fluids infused in both the CF‐PCV and CF‐PSV groups were multiplied by 4 for the cumulative amount of fluid for LF; thus, the infusion rate was adjusted for the LF‐PSV and LF‐PCV groups for 1 h (*n* = 6 per group). The pilot procedure did not hinder the randomization of the animals. The study design and temporal evolution are summarized in Figure 1. At *T*_60_, heparin was injected (1000 U i.v.), and the animals were euthanized by an intravenous overdose of sodium thiopental (60 mg/kg; Cristália). The trachea was clamped at PEEP = 3 cmH_2_O, and the lungs and kidneys were removed *en bloc* for histology and molecular biology analysis.

### 2.4 Data acquisition and respiratory mechanics

Airflow, airway pressure (*P*_aw_), and esophageal pressure (*P*_es_) were recorded throughout the experiments by software written in LabView (National Instruments) (Wierzchon et al., 2017). V_T_ was calculated by digital integration of the airflow signal. Respiratory rate was calculated from swings in *P*_es_ as the frequency per minute of each type of breathing cycle. Transpulmonary pressure (*P*_L_) was calculated during inspiration and expiration as the difference between *P*_aw_ and *P*_es_. Peak and plateau transpulmonary pressures (*P*_peak,L_ and *P*_plat,L_) were measured. To calculate the plateau pressures (*P*_plat_), the airways were occluded at end inspiration (zero airflow) for 3 s. Static transpulmonary driving pressure (Δ*P*_Lstat_) was calculated at the end of inspiration (max) and expiration (min) as the difference between driving airway pressure (Δ*P*_RS_) and the change in esophageal pressure (Δ*P*_es_), according to the following equation (Bellani et al., 2016): Δ*P*_L_ = Δ*P*_RS_ − Δ*P*_es_ where Δ*P*_es_ = *P*es_max_ − *P*es_min_. *P*oes_0.1_ is the esophageal pressure measured at 100 ms of inspiration after occlusion at end expiration, which reflects the neuromuscular drive (Elliott et al., 1993). All signals were amplified in a 4‐channel signal conditioner (SC‐24; SCIREQ) and sampled at 200 Hz with a 12‐bit analog‐to‐digital converter (National Instruments). All data were computed by a routine written in MATLAB (R2007a; MathWorks).

### 2.5 Echocardiography

Transthoracic echocardiography was performed (UGEO HM70A, 8–13 MHz transducer; Samsung) at *T*_0_ and *T*_60_ according to the American Society of Echocardiography recommendations (Lang et al., 2015). The following M‐mode, bidimensional, and pulsed Doppler dicom images were captured and later analyzed in EchoPac (GE Healthcare): the diameter of the internal vena cava (IVC_d_), velocity‐time integral (VTI), aortic valve diameter (AV_d_), and pulmonary acceleration to pulmonary ejection time ratio (PAT/PET). Left ventricle cardiac output (CO_LV_) was calculated as [(AV_d_)^2^ × 0.785 × (VTI)] × (heart rate); variation in left ventricle stroke volume SVV_LV_ as (SV_max_ − SV_mín_) × 100/[(SV_máx_ + SV_mín_)/2]; and the vena cava collapsibility index (VCCI) as ((IVC_dmax_ − IVC_dmin_) × 100/IVC_dmin_).

### 2.6 Lung histology

#### 2.6.1 DAD score

All pathologists were blinded to the group assignment. To assess the diffuse alveolar damage (DAD) score, the left lung was embedded in paraffin after fixation in 4% formaldehyde. Sections were cut 4 μm thick longitudinally from the central zone with a microtome and stained with hematoxylin‐eosin (HE). The left lung was assessed in a light microscope (Olympus BX51; Olympus Latin America) for the DAD score by two independent investigators (A.C.F.F. and V.L.C.) blinded to group assignment. The scores of each expert were combined to yield a final score by arithmetic averaging (Kiss et al., 2016). The value of kappa was 0.82. Photomicrographs at magnifications of ×100, ×200, and ×400 were obtained from 10 non‐overlapping fields of view per section using a light microscope (Olympus BX51, Olympus Latin America). Briefly, scores of 0–4 were used to represent the severity of edema and inflammatory infiltrate in both axial and alveolar parenchyma, thus totaling four parameters (Uhlig et al., 2014). In addition, the extent of each scored characteristic per field of view was determined on a scale of 0–4, with 0 denoting no visible evidence and 4 denoting widespread involvement. Scores were calculated as the product of the severity and extent of each parameter on a range of 0–16. The cumulative DAD score was calculated as the sum of each parameter characteristic and ranged from 0 to 64.

#### 2.6.2 Quantification of heterogeneous airspace enlargement

Airspace enlargement was assessed by measuring the mean linear intercept between alveolar walls at a magnification of ×400, as described elsewhere (Hsia et al., 2010). The central moment of the mean linear intercept (*D*_2_ of the mean linear intercept between the alveolar walls) was calculated from 20 airspace measurements (Parameswaran et al., 2006) using the following equation:

$$D_{2}=\mu \cdot \left(1+\frac{\sigma^{2}}{\mu^{2}+\sigma^{2}}\right)\cdot \left(2+\sigma \cdot \frac{\gamma }{\mu }\right)$$

where *μ* is the mean, *σ* is the standard variation and *σ* ^2^ is the variance in airspace diameter, and *γ* is the skewness of the diameter dispersion. The heterogeneity index, represented by *β*, was calculated by the ratio between *D*_2_ and the mean linear intercept between the alveolar walls (Wierzchon et al., 2017).

#### 2.6.3 Quantification of perivascular edema

To quantify perivascular edema, 10 random, noncoincident microscopic fields containing vessels were evaluated. The number of points falling on areas of perivascular edema and the number of intercepts between the lines of the integrating eyepiece and the basal membrane of the vessels were counted. The interstitial perivascular edema index was calculated as follows: number of points^0.5^/number of intercepts (Cavalcanti et al., 2014; Santiago et al., 2010).

#### 2.6.4 Quantification of occludin by immunohistochemistry

The specimens for lung immunohistochemistry were obtained from the archived formalin‐fixed paraffin‐embedded lung histologic sections incubated with anti‐occludin monoclonal antibody diluted in 50 parts of citrate (Santa Cruz sc‐133256, Santa Cruz Biotechnology) and were placed in tissue microarrays from most representative areas previously marked in HE‐stained slides. Occludin quantification was measured as the number of positive cells/mm^2^ and performed using a semi‐assisted method in QuPath (v0.2.1; Centre for Cancer Research & Cell Biology, Edinburgh University) as described previously (Balancin, Teodoro, Baldavira, et al., 2020; Balancin, Teodoro, Farhat, et al., 2020).

#### 2.6.5 Immunofluorescence of occludin and claudin‐4

Immunofluorescence was performed on an immunofluorescence microscope (Olympus BX51, Olympus) for qualitative assessment of occludin and claudin‐4 using the following antibodies: anti‐occludin Santa Cruz (sc‐133256, Santa Cruz Biotechnology), anti‐vimentin (D21H3 XP mAb; Cell Signaling, Uniscience do Brasil), anti‐claudin‐4 (ab15104; Abcam), and anti‐actin (M0851, Dako; Agilent Technologies Brasil) diluted 1:30; 1:100; 1:300, and 1:100 in citrate, respectively.

### 2.7 Kidney histology

Two semi‐quantitative score systems were used for kidney histology. Brush border analysis was performed in digital photos of 10 kidney regions from 400× magnified slides stained with periodic acid Schiff. Scores ranged from 0 to 4 according to the area affected by brush border loss, cell tumefaction, and interstitial edema (0, no evidence; 1, 1%–25%; 2, 25%–50%; 3, 50%–75%; 4, 75%–100%). The final score was the mean from 10 visual fields and ranged between 0 (no damage) to 4 (full involvement). The acute kidney injury (AKI) score was adapted from previous studies (Bateman et al., 2016; Hüter et al., 2009) and was measured in HE‐stained kidney slides, ranging from 0% to 100%. Image‐Pro Plus v.6.0.0.260 (Media Cybernetics) was used to calculate the percentage of the visual field corresponding to calibrated colors associated with edema. The results are presented as the mean of 10 visual fields. Semi‐quantitative score was calculated by two expert investigators (A.C.F.F. and V.L.C.) blinded to group assignment.

### 2.8 Molecular biology of lung and kidney

Quantitative real‐time reverse transcription polymerase chain reaction (RT‐qPCR) was performed on kidneys for kidney injury molecule 1, neutrophilic gelatinase‐associated lipocalin (NGAL), and interleukin‐6 (IL‐6), and on lung tissue for zona occludens‐1 (ZO‐1) and metalloproteinase‐9 (MMP‐9). The primer sequences are shown in Table S1. Slices from the right superior lung lobe and right kidney were sampled in cryotubes and stored at −80°C after immersion in liquid nitrogen. Extraction of total RNA was achieved with an RNA total SV system (Promega). RNA concentration was measured using Nanodrop ND‐1000 spectrophotometry. cDNA was synthesized and amplified from total RNA with a GoTaq 2‐STEP RT‐qPCR system (Promega). The RT‐PCR reaction was performed with Applied Biosystems SYBR green PCR Master Mix (Thermo Fisher Scientific), and relative mRNA was measured using an SYBR green detection system (ABI 7500 Real‐Time PCR; Applied Biosystems). For each sample, the expression of each gene was normalized to that of the housekeeping gene 36B4 (Akamine et al., 2007) and expressed as fold change relative to the nonventilated group, using the 2^−∆∆Ct^ method, where ΔCt = Ct (reference gene) − Ct (target gene). All analyses were performed by M.A.A. who was blinded to the group assignment.

### 2.9 Statistical analysis

Sample size was calculated using G*Power 3.9.1.2 (Düsseldorf University, Düsseldorf, Germany) and the following settings: power (1 − *β* = 0.8); significance level (*α* = 0.05); allocation ratio N2/N1 = 1; effect size (*d* = 1.83) according to the difference in the DAD score between PSV and PCV obtained from published data (Magalhães et al., 2018). Comparisons were made between groups under the same ventilatory or fluid strategy (CF‐PCV vs. CF‐PSV; CF‐PCV vs. LF‐PCV; CF‐PSV vs. LF‐PSV; LF‐PCV vs. LF‐PSV). The primary outcome was the DAD score, and the secondary outcomes were alveolar heterogeneity, expression of occludin in lung tissue, as well as expression of claudin‐4, vimentin, and α‐actin in lung tissue, and molecular biology data. Data were tested for normality by the Shapiro–Wilk test. Body weight, the alveolar heterogeneity index, AKI score, and brush border analysis were assessed by one‐way ANOVA with Holm‐Sidak multiple comparison tests. Fluid composition, respiratory mechanics, and hemodynamics were assessed by two‐way ANOVA with Holm‐Sidak multiple comparison tests. Fluid infusion rate, molecular biology data, perivascular edema, DAD score, and quantification of occludin were compared using the Kruskal–Wallis test followed by Dunn's multiple comparison tests. Spearman correlation was calculated for MAP versus CO_LV_, fluid infusion rate versus perivascular edema, fluid infusion rate versus DAD, and occludin versus DAD. Parametric data were expressed as the mean ± *SD* and nonparametric data as the median (interquartile range). All tests were performed in GraphPad 9 (GraphPad Software).

## 3 RESULTS

All animals survived to the end of the experiment, without any missing data. LF‐PCV and LF‐PSV received a total amount of fluid (median [interquartile range]) equal to 23.1 ml/kg/h (22.7–24.8) and 26.5 ml/kg/h (23.2–34.8), respectively; CF‐PCV and CF‐PSV received at total amount of fluid equal to 4.2 ml/kg/h (3.5–9.1) and 4.4 ml/kg/h (3.1–8.0), respectively, as shown in Figure S1.

No significant differences were observed in MAP and CO_LV_ among the groups. SVV_LV_ and VCCI were lower in the FL‐PCV group at *T*_60_ compared with *T*_0_ (*p* = 0.023 and *p* = 0.037, respectively), showing hypervolemia status (Table S2). All groups were protectively ventilated (V_T_ = 6 ml/kg), and no difference in static Δ*P*_L_ was found among the groups. *P*_peak,L_ and *P*_plat,L_ were higher in both PSV groups compared with the PCV groups, regardless of the fluid regimen (Table S3). No significant difference in *P*oes_0.1_ was observed between the CF‐PSV and LF‐PSV groups.

**FIGURE 2:**
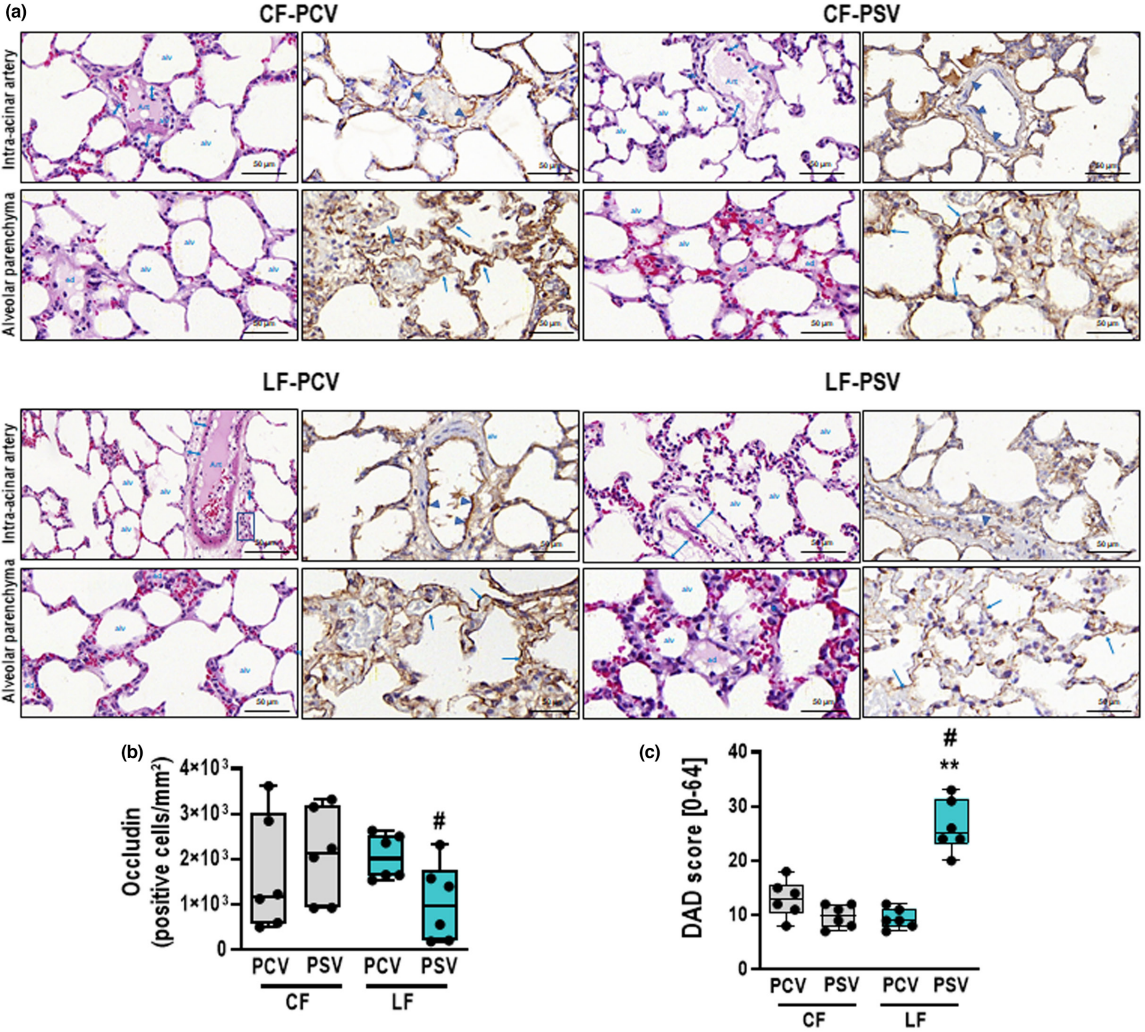
DAD score and immunohistochemistry for occludin. (a) Representative images of histologic sections of alveolar pulmonary parenchyma (alveolar sacs and ducts, alveoli, and associated capillary loops) and perivascular sections (intra‐acinar arteries) stained by hematoxylin‐eosin and immunohistochemistry for occludin at high magnification. The intra‐acinar artery sections of CF‐PCV demonstrate discrete cuff‐shaped perivascular edema (double blue arrowheads) coincident with prominent expression of occludin by the endothelial cells of the artery (blue arrowheads). A similar immunophenotypic pattern of occludin was found in CF‐PSV and LF‐PCV, except for slightly cuff‐shaped perivascular edema (blue arrowheads) and mild inflammatory cells (blue square). In contrast, the LF‐PSV alveolar parenchyma exhibited almost absence of occludin in the endothelium of capillary loops (blue arrows). (b) Boxplot of occludin quantification in lung tissue. *p* = 0.026. (c) DAD score. **, means different from CF‐PSV (*p* < 0.01); #, vs LF‐PCV (*p* < 0.05). Alv, alveoli; art, artery; CF, conservative fluid therapy; DAD, diffuse alveolar damage; ed, edema; LF, liberal fluid therapy; PCV, pressure‐controlled ventilation; PSV, pressure‐support ventilation.

**FIGURE 3:**
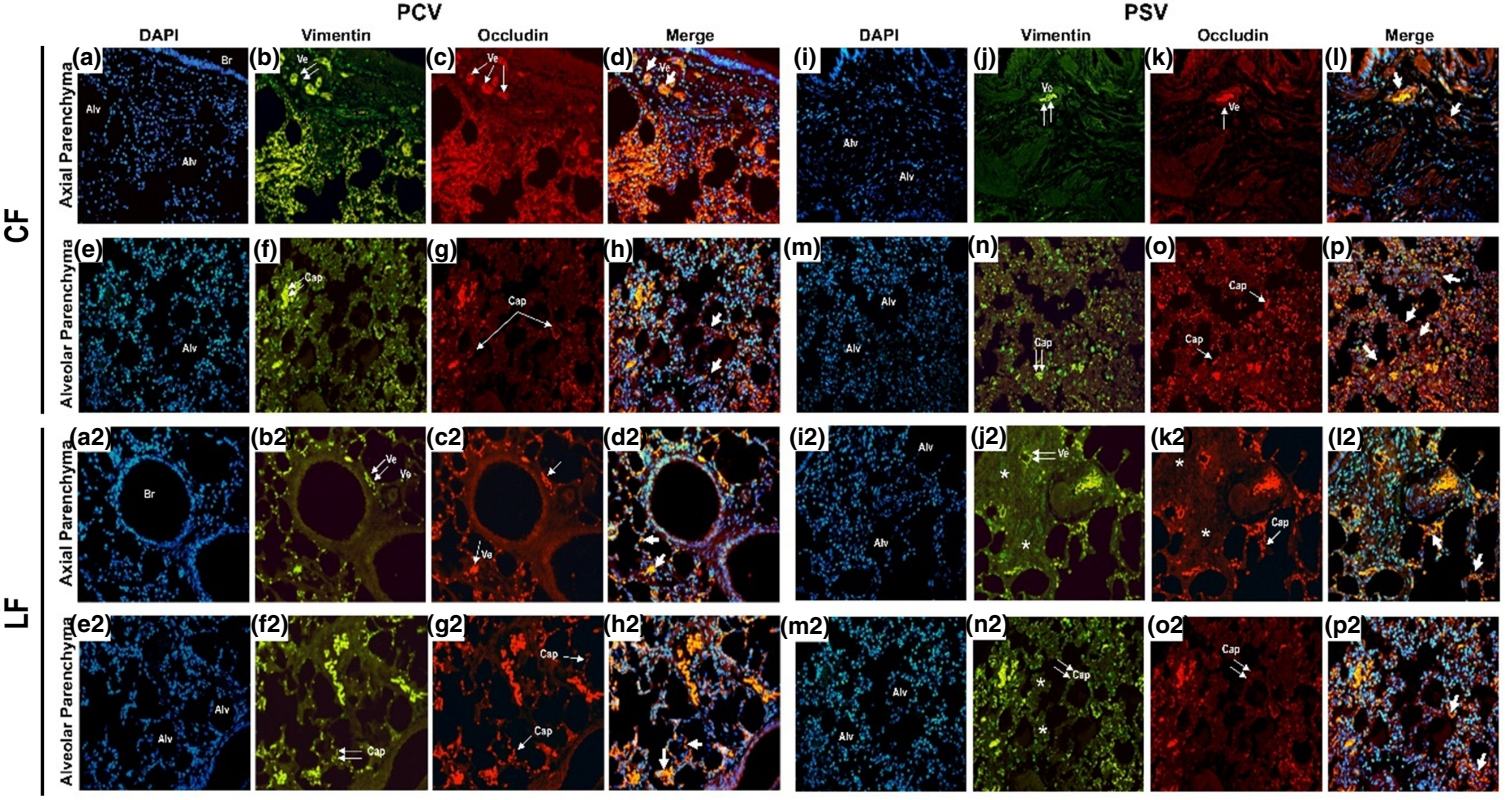
Immunofluorescence of occludin and vimentin. Histologic sections (×400) of the axial and alveolar staining for immunofluorescence to occludin and vimentin divided by groups (CF‐PCV, CF‐PSV, LF‐PCV, and LF‐PSV). The arrows indicate the endothelial cells of the bronchiolar and alveolar capillaries expressing occludin [nuclei in blue, 4′,6‐diamidino‐2‐phenylindole (DAPI); connective tissue in green; vimentin; bronchiolar vessels and alveolar capillaries in red; occludin and colocation, blue‐orange‐red]. (a) DAPI (nuclei in blue) in axial parenchyma in CF‐PCV. (b,f) Vimentin in axial parenchyma in CF‐PCV. (d) merge of DAPI, vimentin and occludin in axial parenchyma in CF‐PCV. (e) DAPI (nuclei in blue) in alveolar parenchyma in CFPCV. (f) Vimentin in alveolar parenchyma in CF‐PCV. (h) Merge of DAPI, vimentin and occludin in alveolar parenchyma in CF‐PCV. (i) DAPI (nuclei in blue) in axial parenchyma in CF‐PSV. (j) Vimentin in axial parenchyma in CF‐PSV. (l) Merge of DAPI, vimentin and occludin in alveolar parenchyma in CF‐PCV. (m) DAPI (nuclei in blue) in alveolar parenchyma in CF‐PSV. (n) vimentin inalveolar parenchyma in CF‐PSV. (p) Merge of DAPI, vimentin and occludin in alveolar parenchyma in CF‐PSV. The CF‐PCV parenchyma (C and G) presents intense red birefringence of occludin in dilated lymphatics and blood vessels around the bronchiolar mucosa and along the capillary alveoli (simple white arrows). CF‐PSV and LF‐PCV (K, O, C2, and G2) present a similar pattern of birefringence for occludin (red) in endothelial cells of the lymphatics and blood vessels (double white arrows), as well as along capillaries of the alveolar septa (single white arrows). In contrast, LF‐PSV (K2 and O2) has almost no birefringence of occludin in the endothelium of the lymphatics and blood vessels, as well as along capillaries of the alveolar septa, with prominent oedema in this group (*). Br, bronchioles; Alv, alveoli; Ve, blood vessels; Cap, capillaries. White double arrows, connective tissue around bronchiolar vessels and alveolar capillaries. Simple white arrows, expression of occludin by endothelial cells of bronchiolar vessels and alveolar capillaries; white arrowhead, colocation of vimentin and occludin; *, peribronchiolar and alveolar cuff.

The alveolar heterogeneity index was similar among the groups (Table 1). The cumulative DAD score was lower with CF‐PSV and LF‐PCV than LF‐PSV, mainly due to lower axial parenchyma and intra‐alveolar parenchyma edema (*p* < 0.05 for both) (Figure 2; Table 1). Axial parenchyma edema, intra‐alveolar edema, and cumulative DAD score were positively correlated with the fluid infusion rate (*r* = 0.544; *r* = 0.600, and *r* = 0.494, respectively, with *p* < 0.05 for all three correlations) (Figure S2). The perivascular edema index was lower in both CF groups compared with the LF groups (CF‐PCV vs. LF‐PCV, *p* = 0.046; CF‐PSV vs. LF‐PSV, *p* = 0.009), and positively correlated with the fluid infusion rate (*r* = 0.424, *p* = 0.039). LF‐PCV was associated with increased expression of occludin in lung tissue compared with LF‐PSV (*p* = 0.026, Figure 2). The expression of occludin in lung tissue was negatively associated with axial and intra‐alveolar parenchyma edema (*r* = −0.432 and *r* = −0.438, respectively; *p* < 0.05 for both correlations). Qualitative assessment of occludin and claudin‐4 by immunofluorescence showed increased birefringence for the LF‐PCV group compared with the LF‐PSV group (Figure 3; Figure S3, respectively).

**TABLE 1:** Cumulative diffuse alveolar damage score and its components, alveolar heterogeneity index, and perivascular edema

	CF		LF	
	PCV	PSV	PCV	PSV
Axial parenchyma				
Edema (cuff) (0–16)	2 (1–4)	3 (1–4)	2 (2–2.5)	10 (7–12)^#^ ^,^ ^†^
Inflammatory infiltrate (0–16)	3.5 (3–6.8)	3 (1.75–4)	4 (3.8–4.5)	3 (2–4)
Alveolar parenchyma				
Edema intra‐alveolar (0–16)	3 (1–4)	1 (0–2)	1.5 (0.8–2.5)	10.5 (8–13)^#^ ^,^ ^†^
Inflammatory infiltrate (0–16)	3.5 (2.8–4)	4 (2–4)	2.5 (1–4)	3 (2–3.8)
Cumulative DAD score (0–64)	13 (10.3–15.8)	10 (7.8–12)	9 (7.8–11.3)	25 (23–31.5)^#^ ^,^ ^†^
*β* (*D*_2_/Lm)	1.29 (1.21–1.42)	1.26 (1.19–1.50)	1.32 (1.24–1.52)	1.20 (1.07–1.31)
Perivascular edema	0.62 (0.54–0.68)	0.57 (0.52–0.62)	0.83 (0.63–0.85)*	0.71 (0.64–0.84)^#^

*Note*: Values are presented as the median (interquartile range [25%–75%]) of 6 animals per group. Comparisons were performed by Kruskal–Wallis test followed by Dunn's multiple comparison test (*p* < 0.05).

Abbreviations: CF, conservative fluid therapy; DAD, diffuse alveolar damage; LF, liberal fluid therapy; PCV, pressure‐controlled ventilation; PSV, pressure‐support ventilation; β, alveolar heterogeneity index.

* *p* < 0.05 significantly different from CF‐PCV.

^#^ *p* < 0.05 significantly different from CF‐PSV.

^†^ *p* < 0.05 significantly different from LF‐PCV.

**FIGURE 4:**
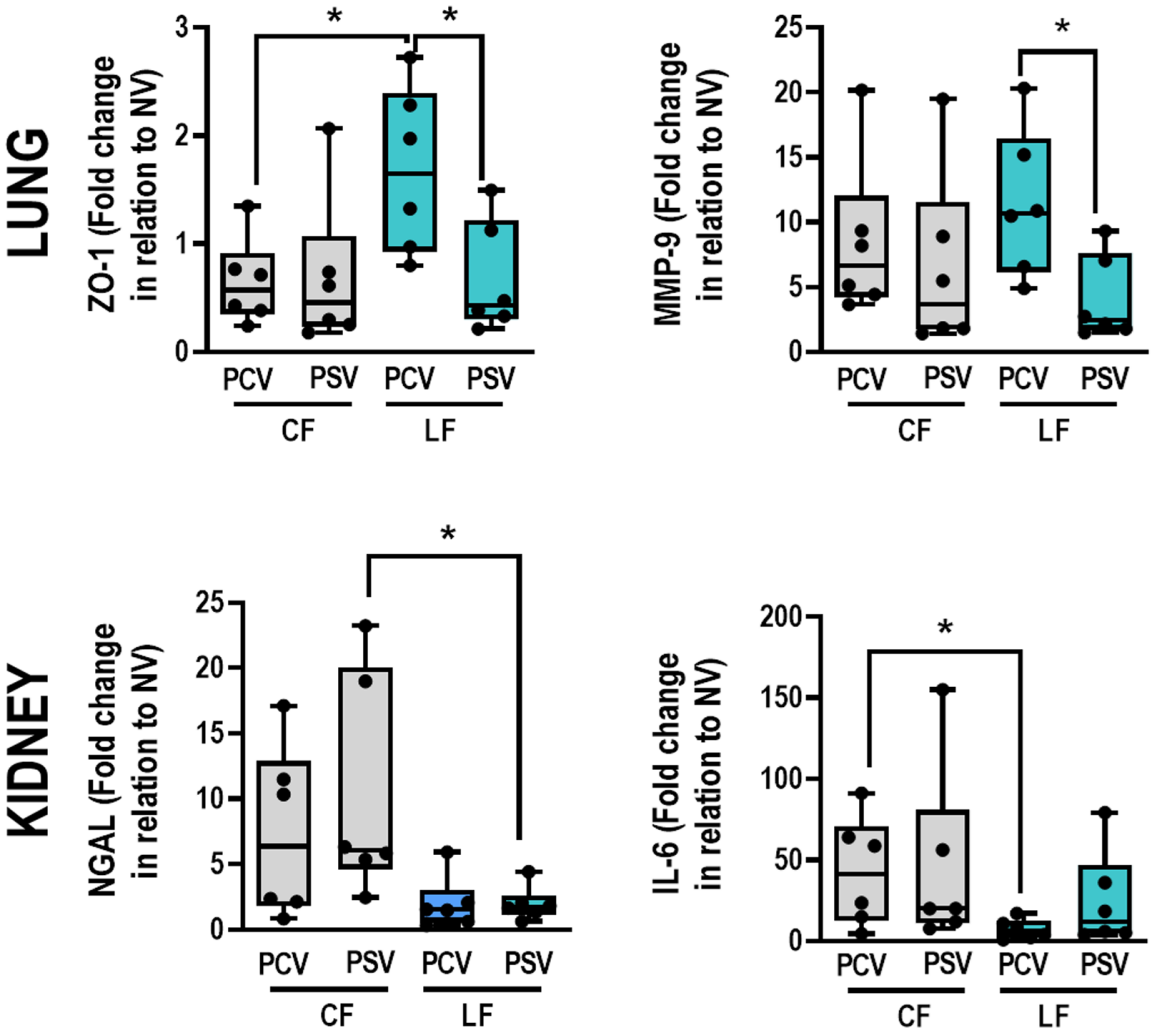
Molecular biology of lung and kidney. Gene expression of ZO‐1 and MMP‐9 in lung tissue; and KIM‐1, NGAL, and IL‐6 in kidney tissue. Boxplots represent the median and interquartile range of 6 animals per group. Comparisons were done by Kruskal–Wallis test followed by Dunn's multiple comparisons test (*p* < 0.05). CF, conservative fluid therapy; IL‐6, interleukin‐6; KIM‐1, kidney injury molecule 1; LF, liberal fluid therapy; MMP‐9, metalloproteinase‐9; NGAL, neutrophilic gelatinase‐associated lipocalin; PCV, pressure‐controlled ventilation; PSV, pressure‐support ventilation; ZO‐1, zona occludens‐1; *, depicting the differences between groups (*p* < 0.05).

In lungs, ZO‐1 gene expression was higher in the LF‐PCV group than in the LF‐PSV group (*p* = 0.041) and increased more in the LF‐PCV group than in the CF‐PCV group (*p* = 0.015). MMP‐9 expression was lower in the LF‐PCV group than in the LF‐PSV group (*p* = 0.026) (Figure 4).

In kidneys, gene expressions of NGAL and IL‐6 did not differ significantly between the LF‐PSV and LF‐PCV groups. NGAL expression was lower in the LF‐PSV group than in the CF‐PSV group (*p* = 0.021). IL‐6 expression was reduced more in the LF‐PCV group than in the CF‐PCV group (*p* = 0.026). In addition, no significant differences in AKI scores and brush border analysis were detected between the different groups (Table S3).

## 4 DISCUSSION

In the present ALI model, we found that ALI compared with LF during pressure support ventilation: (1) the DAD score decreased more, mainly due to edema in axial and alveolar parenchyma; (2) birefringence for occludin and claudin‐4 in lung tissue was increased; and (3) neutrophil gelatinase‐associated lipocalin expression in kidney tissue increased more. Pressure support ventilation in combination with CF, but not LF, promoted less lung epithelial damage in this model of ALI. However, the beneficial effects on kidney were observed in LF compared with ALI. Our data suggest that the fluid strategy should be strictly monitored during mechanical ventilation, mainly during pressure support ventilation.

We used a model of *E. coli* LPS induced‐mild lung injury because it reproduces changes in lung function and histology (alveolar collapse, neutrophil infiltration, and edema) of human ARDS (Matute‐Bello et al., 2011). A mean fluid infusion rate as low as 5.4 ml/kg/h (observed in the CF‐PCV and CF‐PSV groups) was sufficient to maintain MAP ≥70 mmHg and normal CO_LV_ (Watson et al., 2004) values in the CF groups, probably because intratracheal instillation of LPS, as opposed to sepsis‐induced lung injury, does not produce severe hemodynamic instability (Famous et al., 2017). Similar results have been shown previously by our group (Magalhães et al., 2018; Moraes et al., 2014, 2018; Rocha et al., 2021; Saddy et al., 2013). We defined the LF rate as four times the CF rate because there is a lack of consensus on what a “liberal” fluid strategy means in clinical practice (Rahbari et al., 2009). Nevertheless, the difference between the CF and LF infusion rates was close to that in many other studies (Holte et al., 2007; Kotlińska‐Hasiec et al., 2017; Lobo et al., 2011; Shin et al., 2018). Even though CO_LV_ and MAP were similar between the groups and time points, the fluid rate difference was sufficient to create two different hemodynamic profiles as demonstrated by lower SVV_LV_ and VCCI at *T*_60_ in the LF‐PCV group. These results suggest a fluid overload (corresponding to Frank‐Starling's curve plateau) that could potentially increase the hydrostatic pressure in pulmonary capillaries. All groups were successfully ventilated under protective V_T_ (≈6 ml/kg) and had similar alveolar heterogeneity indexes as previously reported (Pinto et al., 2020).

Although perivascular edema was positively correlated with higher fluid infusion rates (i.e., higher in the LF groups regardless of the ventilatory strategies), edema in axial and alveolar lung parenchyma yielded higher cumulative DAD scores in the LF‐PSV group. The LF‐PSV group had higher edema scores than reported in previous studies not combining LFs and spontaneous breathing (Magalhães et al., 2018; Moraes et al., 2018; Pinto et al., 2020; Silva et al., 2018). We can explain the increase in axial and alveolar edema and ultimately the DAD score in the LF‐PSV group as follows: (1) under an LF strategy, an increase in hydrostatic capillary pressure and filtration is expected, mainly at increased lung permeability, as caused by LPS (Pugin et al., 1999; Ware & Matthay, 2000); (2) during PSV, transpulmonary pressure is determined by increased airway pressure and decreased pleural pressure, leading to tensile stress (Bellani et al., 2016; Saddy et al., 2014), which in turn, may stretch alveolar units through repetitive movements; and (3) edema may increase further if tight junction connections, which are constitutive in epithelial and endothelial structural cells, are lost during the stretch movements produced by tensile stress in PSV (Cavanaugh Jr et al., 2001). In addition, the level of neuromuscular drive (*P*oes_0.1_) in CF or LF groups during PSV was similar to other studies in small animals with ALI (da Cruz et al., 2021). We hypothesize that injury in tight junctions has a mechanistic relationship with fluid overload because expression of occludin was lower in the LF‐PSV group and negatively correlated with edema with lower birefringence for claudin‐4. Similar behavior was not observed with the CF strategy in combination with pressure support ventilation. This suggests that the application of pressure support ventilation during early hemodynamic resuscitation may worsen lung injury.

LF‐PSV had lower expression of ZO1 than LF‐PCV, suggesting less tight junction recovery and higher cell proliferation with suppression of apoptosis (Meng et al., 2020; Ogata‐Suetsugu et al., 2017). Lower MMP‐9 gene expression with LF‐PSV than with LF‐PCV has already been described in a similar model of acute respiratory distress syndrome receiving approximately 10 ml/kg/h of fluids (Pinto et al., 2020), a difference not observed under a CF strategy.

Kidney histology indicates that both CF and LF were not associated with increased injury. It is possible that the AKI score and brush border analysis revealed almost no impact because of the short time span of the intervention. Kidney gene expression was similar between the groups, although in a post‐hoc analysis, both LF‐PCV and LF‐PSV groups had significantly lower expression of NGAL and IL‐6 than in the CF groups. This suggests that the CF strategy may be associated with some degree of kidney injury, although we did not analyze urine or blood markers of kidney function.

### 4.1 Limitations

The study has some limitations to be addressed. First, the results apply to this specific model of ALI and cannot be directly extrapolated to the clinical scenario. However, as extensively discussed previously for LPS models (Cardinal‐Fernández et al., 2017; Matute‐Bello et al., 2008), the DAD scores presented here confirm ALI just as in previous studies. The histologic features better represent mild lung injury from LPS instillations in small animals (Matute‐Bello et al., 2011). Second, the LF rates used here were relatively high; nevertheless, studies show similar fluid rates in clinical scenarios (Holte et al., 2007; Kotlińska‐Hasiec et al., 2017; Lobo et al., 2011; Shin et al., 2018). Furthermore, we aimed to investigate groups with extreme and different fluid strategies. Third, due to the study design, only the PCV group required neuromuscular blockade, and these drugs may reduce inflammatory responses in the setting of ALI (Forel et al., 2006). Fourth, whether a higher PEEP strategy would protect from the effects of pressure support ventilation and LF management on pulmonary edema remains unknown. Fifth, although the time under mechanical ventilation is relatively short, it should be normalized to the metabolism of small mammals whereby 1 rat hour approximates 27 human hours (Agoston, 2017), and specifically to VILI progression, it should be normalized to breathing frequency (6‐ to 7‐fold higher in rats than humans). Nevertheless, the 1‐h experiment was time enough to observe decrements in constitutive proteins at the alveolar‐capillary membrane and changes in gene expression.

## 5 CONCLUSIONS

CF compared with LF during pressure support ventilation yielded less lung epithelial cell damage in the current model of ALI. However, LF compared with conservative fluid resulted in less kidney injury markers, regardless of the ventilatory strategy.

## AUTHOR CONTRIBUTIONS

This study was conducted at the Pulmonary Investigation Laboratory in Institute of Biophysics Carlos Chagas Filho in Federal University of Rio de Janeiro. Eduardo Butturini de Carvalho, Marcelo Gama de Abreu, Nathane Santanna Felix, Paolo Pelosi, Patrícia Rieken Macêdo Rocco, and Pedro Leme Silva were involved in the conception or design of the work; Eduardo Butturini de Carvalho, Ana Carolina Fernandes Fonseca, Raquel Ferreira Magalhães, Eliete Ferreira Pinto, Cynthia dos Santos Samary, Mariana Alves Antunes, Camila Machado Baldavira, Lizandre Keren Ramos da Silveira, Walcy Rosolia Teodoro, Vera Luiza Capelozzi, and Pedro Leme Silva took part in the acquisition, analysis or interpretation of data; Eduardo Butturini de Carvalho, Marcelo Gama de Abreu, Nathane Santanna Felix, Paolo Pelosi, Patrícia Rieken Macêdo Rocco, and Pedro Leme Silva drafted the work or revised it critically for important intellectual content. All authors approved the final version of the manuscript, agree to be accountable for all aspects of the work, ensuring that questions related to the accuracy or integrity of any part of the work are appropriately investigated and resolved. All persons designated as authors qualify for authorship, and all those who qualify for authorship are listed.

## FUNDING INFORMATION

This study was supported by the Brazilian Council for Scientific and Technological Development (CNPq; 483005/2020‐6), the Rio de Janeiro State Research Foundation (FAPERJ), São Paulo Research Foundation (FAPESP; 2018/20403‐6, 2019/12151‐367 0), and Coordination for the Improvement of Higher Education Personnel (CAPES) (CAPES; Finance Code 001), and the Department of Science and Technology ‐ Brazilian Ministry of Health (DECIT/MS).

## CONFLICT OF INTEREST

The authors declare no competing interests.

## Supporting information


Table S1

Table S2

Table S3

Figure S1

Figure S2

Figure S3
