# Supplementary material for: Effects of different fluid management on lung and kidney during pressure‐controlled and pressure‐support ventilation in experimental acute lung injury
Source: Physiol Rep. 2022 Sep 6;10(17):e15429. doi: 10.14814/phy2.15429 (PMC9446390; doi:10.14814/phy2.15429)
Supplement: Supplementary file 1 — Table S1 [file PHY2-10-e15429-s004.docx]

**Supplemental Table S1**

| **Gene** | **Primer** | **Sequence (5′–3′)** |
| --- | --- | --- |
| 36B4 | F | AAT CCT GAG CGA TGT GCA G |
|  | R | GCT GCC ATT GTC AAA CAC |
| IL-6 | F | CTC CGC AAG AGA CTT CCA G |
|  | R | CTC CTC TCC GGA CTT GTG A |
| KIM-1 | F | GAA GAA AAC AAAT GGA TCA AGG GAT T |
|  | R | GGA GTG GAA ATG GCT CTA ATG AAC T |
| NGAL | F | TTG GGA CAG GGA AGA ACG A |
|  | R | TCA ACG CTG GGC AAC ATT A |
| Amphiregulin | F | AAG AAT CCG TGT GCC GCC AAG TTT |
|  | R | TTT CTC CAC ACC GTT CGC CAA AGT |
| ANGPT-2 | F | CAG CCA ACC AGG TGA TT |
|  | R | AAG TTG GAA GGA CCA CAT GC |
| MMP9 | F | CCA CCG AGC TAT CCA CTC AT |
|  | R | GTC CGG TTT GAG CAT GTT TT |
| TNF-α | F | TTG CTT CTT CCC TGT TCC |
|  | R | CTG GGC AGC GTT TAT TCT |
| ZO1 | F | CAC CAC AGA CAT CCA ACC AG |
|  | R | CAC CAA CCA CTC TCC CTT GT |
