# Supplementary material for: Effects of different fluid management on lung and kidney during pressure‐controlled and pressure‐support ventilation in experimental acute lung injury
Source: Physiol Rep. 2022 Sep 6;10(17):e15429. doi: 10.14814/phy2.15429 (PMC9446390; doi:10.14814/phy2.15429)
Supplement: Supplementary file 2 — Table S2 [file PHY2-10-e15429-s005.docx]

**Supplemental Table S2**. Hemodynamic, respiratory mechanics and blood gas analysis results

|  |  | **Conservative Fluids (CF)** | | **Liberal Fluids (LF)** | |
| --- | --- | --- | --- | --- | --- |
|  |  | **PCV** | **PSV** | **PCV** | **PSV** |
| MAP (mmHg) | T0 | 129±29 | 124±22 | 122±18 | 127±12 |
|  | T60 | 117±13 | 108±20 | 122±13 | 109±14 |
| CO_LV_ (mL/min) | T0 | 76±25 | 45±11 | 40±22 | 73±42 |
|  | T60 | 67±46 | 70±28 | 52±17 | 67±36 |
| SVV_LV_ (%) | T0 | 55±16 | – | 58±23 | – |
|  | T60 | 54±7 | – | 35±20^†^ | – |
| VCCI (%) | T0 | 22±7 | – | 29±9 | – |
|  | T60 | 18±9 | – | 11±7^†^ | – |
| PaO_2_/FiO_2_ | T0 | 508 ± 94 | 432 ± 160 | 469 ± 90 | 441 ± 154 |
|  | T60 | 529 ± 126 | 537 ± 88 | 472 ± 131 | 455 ± 98 |
| pH | T0 | 7.38 ± 0.10 | 7.40 ± 0.03 | 7.34 ± 0.07 | 7.37 ± 0.04 |
|  | T60 | 7.34 ± 0.06 | 7.37 ± 0.04 | 7.29 ± 0.06 | 7.36 ± 0.54 |
| PaCO_2_ (mmHg) | T0 | 47 ± 20 | 42 ± 7 | 48 ± 13 | 41 ± 4 |
|  | T60 | 44 ± 9 | 43 ± 10 | 52 ± 10 | 38 ± 14 |
| HCO_3_^−^ (mEq/L) | T0 | 25 ± 5 | 27 ± 4 | 24 ± 3 | 24 ± 2 |
|  | T60 | 24 ± 2 | 24 ± 4 | 23 ± 1 | 22 ± 3 |
| V_T_ (mL/kg) | T0 | 6.4 ± 0.8 | 5.6 ± 0.3 | 6.0 ± 0.7 | 6.4 ± 0.8 |
|  | T60 | 5.9 ± 0.5 | 6.4 ± 0.6 | 5.7 ± 1.0 | 6.5 ± 0.6 |
| RR (bpm) | T0 | 55 ± 17 | 80 ± 18 | 54 ± 13 | 70 ± 11 |
|  | T60 | 57 ± 11 | 78 ± 25 | 56 ± 6 | 66 ± 10 |
| P_peak.L_ (cm H_2_O) | T0 | 9.6 ± 1.7 | 15.5 ± 1.6* | 7.9 ± 1.2 | 13 ± 2.7# |
|  | T60 | 9.4 ± 1.0 | 16.3 ± 2.7* | 9.1 ± 1.2 | 13 ± 2.5# |
| P_plat.L_ (cm H_2_O) | T0 | 6.3 ± 0.7 | 12.1 ± 1.8* | 6.9 ± 2.2 | 11.8 ± 2.1# |
|  | T60 | 7.0 ± 1.6 | 13.3 ± 2* | 6.6 ± 2.3 | 11.9 ± 1.8# |
| ΔP_Lstat_ (cm H_2_O) | T0 | 4.5 ± 2.1 | 4.4 ± 0.9 | 6.1 ± 2.5 | 5.3 ± 0.9 |
|  | T60 | 4.5 ± 1.9 | 5.3 ± 1.1 | 6.1 ± 2.4 | 4.7 ± 0.6 |
| Pes_0.1_ (cm H_2_O) | T0 | - | -2,04 ± 0,92 | - | -1,84 ± 0,54 |
|  | T60 | - | -1,33 ± 0,94 | - | -1,62 ± 0,14 |

CF, conservative fluid therapy; LF, liberal fluid therapy; PCV, pressure-controlled ventilation; PSV, pressure-support ventilation; MAP, mean arterial pressure; CO, cardiac output; LV, left ventricle; SVV, stroke volume variation; VCCI, vena cava collapsibility index; PaO_2_/FiO_2_, ratio between oxygen partial pressure in arterial blood and oxygen inspired fraction; PaCO_2_, arterial blood carbon dioxide partial pressure; V_T_, tidal volume; RR, respiratory rate; P_peak.L_, transpulmonary peak pressure; P_plat.L_, transpulmonary plateau pressure; ΔP_Lstat_, static transpulmonary driving; Pes_0.1_, esophageal pressure measured at first 100ms of inspiration.

*Versus CF-PCV (*P*<0.05).

#^1^Versus CF-PCV at respective time point; #^2^versus LF-PCV at respective time point (*P*<0.05); #^3^versus CF-PCV at respective time point; #^4^versus LF-PCV at respective time point (P<0.05).

†Versus respective T0 (*P*<0.05).
