# Supplementary material for: Effects of different fluid management on lung and kidney during pressure‐controlled and pressure‐support ventilation in experimental acute lung injury
Source: Physiol Rep. 2022 Sep 6;10(17):e15429. doi: 10.14814/phy2.15429 (PMC9446390; doi:10.14814/phy2.15429)
Supplement: Supplementary file 3 — Table S3 [file PHY2-10-e15429-s001.docx]

**Supplemental Table S3**. Acute kidney injury score and brush border analysis

|  | **Conservative Fluids (CF)** | | | **Liberal Fluids (LF)** | | |
| --- | --- | --- | --- | --- | --- | --- |
|  | **PCV** | | **PSV** | **PCV** | | **PSV** |
| AKI score | 9.99±1.89 | 9.54±3.37 | | | 7.88±2.97 | 9.82±4.42 |
| Brush border analysis | 0.30±0.37 | 0.20±0.20 | | | 0.42±0.31 | 0.67±0.67 |

AKI and brush border analysis are presented as the mean ± standard deviation of 6 animals per group. Comparisons were performed by one-way ANOVA with Holm-Sidak multiple comparison tests (*P*<0.05). CF, conservative fluid therapy; LF, liberal fluid therapy; PCV, pressure-controlled ventilation; PSV, pressure-support ventilation; AKI, acute kidney injury.
