# Supplementary figures and images for: Effects of different fluid management on lung and kidney during pressure‐controlled and pressure‐support ventilation in experimental acute lung injury
Source: Physiol Rep. 2022 Sep 6;10(17):e15429. doi: 10.14814/phy2.15429 (PMC9446390; doi:10.14814/phy2.15429)

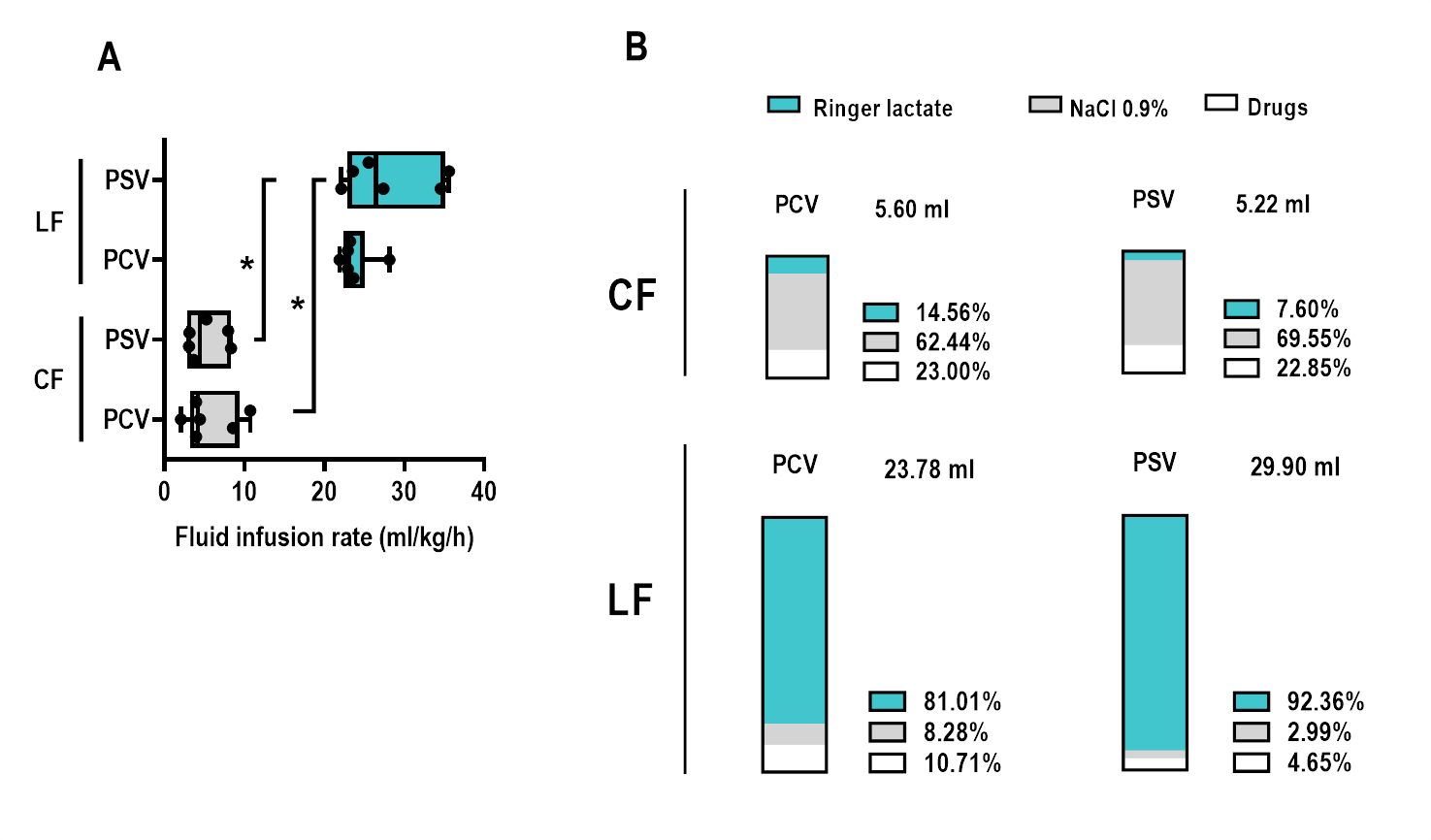

Supplement: Supplementary file 4 — Figure S1 [file PHY2-10-e15429-s002.tif]

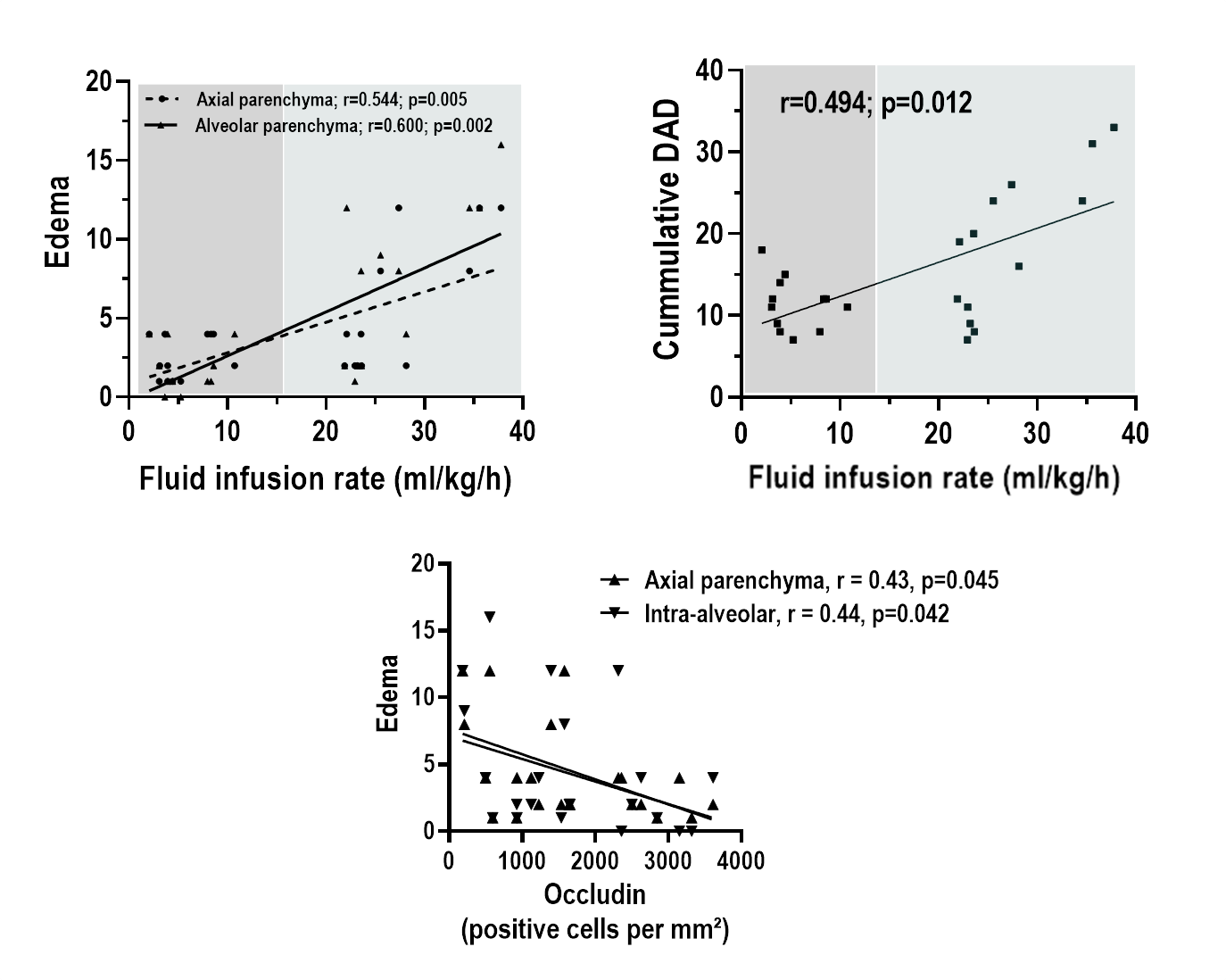

Supplement: Supplementary file 5 — Figure S2 [file PHY2-10-e15429-s006.tif]

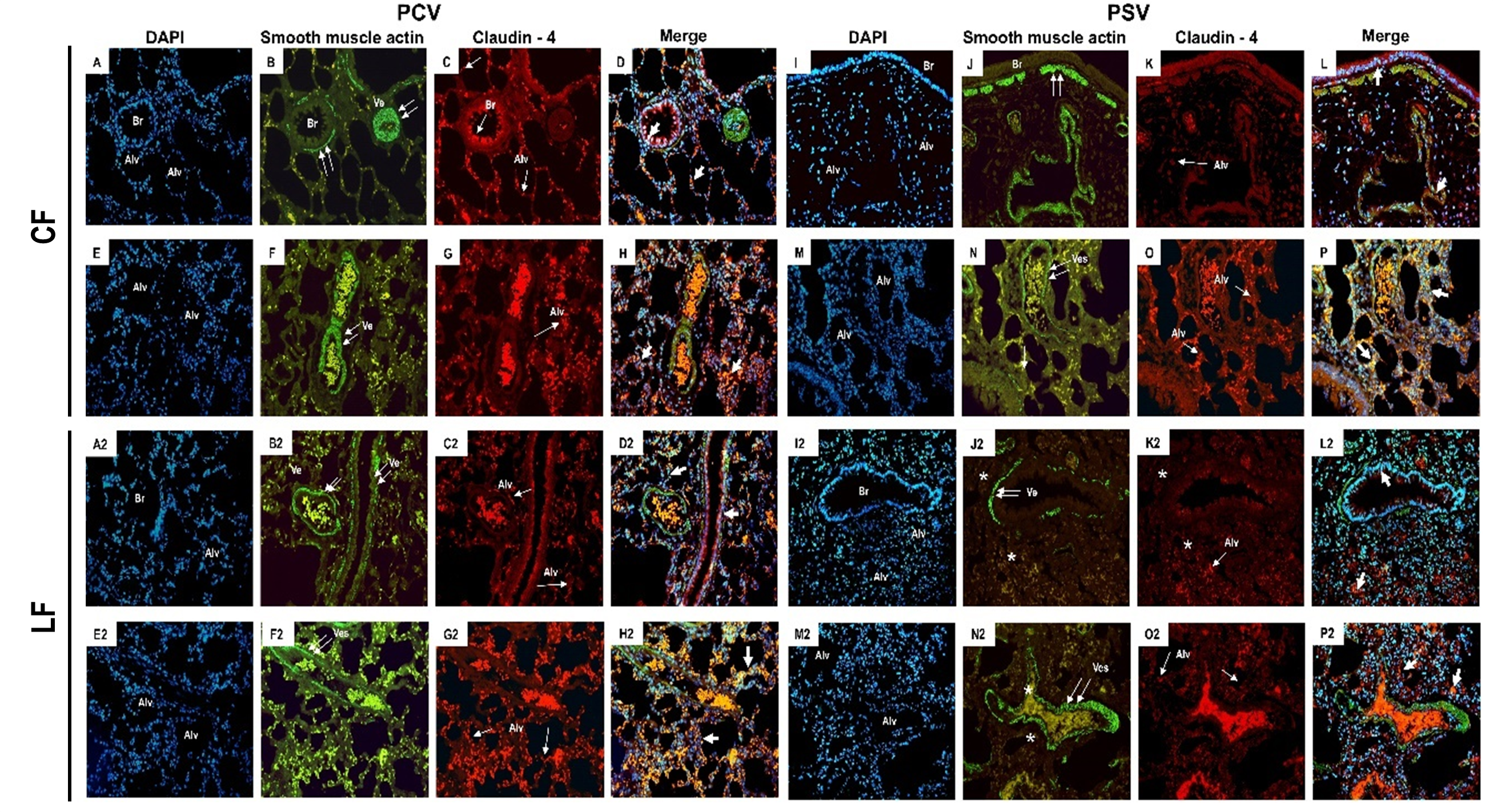

Supplement: Supplementary file 6 — Figure S3 [file PHY2-10-e15429-s003.tif]
